# Human papillomavirus infection rate, distribution characteristics, and risk of age in pre- and postmenopausal women

**DOI:** 10.1186/s12905-021-01217-4

**Published:** 2021-02-25

**Authors:** Yan Shen, Jing Xia, Huihui Li, Yang Xu, Sanping Xu

**Affiliations:** Healthcare Department, Union Hospital, Tongji Medical College, Huazhong University of Science and Technology, Wuhan, China

**Keywords:** Menopausal women, Papillomavirus infection, Cervical cancer screening

## Abstract

**Background:**

The incidence rate of cervical cancer is increasing yearly. The persistent infection of high-risk human papillomavirus (HPV) is the main factor leading to cervical cancer. HPV infection is double peak type. This study aimed at analyzing the HPV distribution characteristics, infection rate, and risk of age in pre- and postmenopausal women. So as to provide reference for the prevention of HPV infection and cervical cancer screening strategy.

**Methods:**

A retrospective analysis of 4614 women who underwent cervical cytology, and HPV examination from January 2018 to October 2019 at the healthcare department of Wuhan Union Hospital was done. We explored the characteristics and distribution of HPV infections around the menopause, then comparing the infection rate of HPV in postmenopause and over 65 years old, in order to analyze the influence of different ages on HPV infection.

**Results:**

Generally, the HPV infection rate was 13.10% (539/4115), whereby the high-risk subtype constituted 73.84% (398/539) of all positive cases. On the other hand, the HPV39 infection was more common in postmenopausal women; however, there was no significant difference in the distribution of the other types in the pre- and postmenopausal women. The first four types were 52/53/58/16. The results further showed that the rates of HPV infection before and after menopause were 12.34% (367/2975) and 15.09% (172/1140), respectively, which had no significant difference (*P* = 0.056), but more susceptible to high-risk HPV infection after the age of 65 (*P* = 0.041). Except for 40 years old to menopause, the infection rate of high-risk HPV in this age group was different from that in postmenopause (*P* = 0.023, 0.729 (0.555, 0.957)), other age groups had no significant effect on high-risk HPV infection.

**Conclusions:**

It was concluded that whether menopause has nothing to do with HPV infection. Moreover, the risk of high-risk HPV infection in women aged 40 to premenopausal is relatively low, but the infection rate increases after 65. Hence the cutoff screening age should be appropriately prolonged.

## Background

Cervical cancer is the second most common cancer in women worldwide, it is estimated that in all over the worldwide about 1.4 million women are living with Cervical cancer (second most after the breast cancer) [1]. Currently, cervical cancer incidence rates present a bipolar pattern at age groups of 35–40 years and 65–80 years in many high-income countries [2]. A study in Costa Rica and Canada also showed a second peak in the infection rate curve for women over 55 [3]. Practically all cervical cancer is caused by high-risk human papillomavirus (h-rHPV) infection [4], and HPV is the most common sexually transmitted virus. Nearly all sexually active people will be infected in their lifetime, some of them may even be repeatedly infected. According to the cross-sectional study, approximately 25–50% of young sexually active women can detect HPV in exfoliated cervical cells or vaginal swab samples, while according to the longitudinal study, this proportion is higher [5].

According to the latest guidelines issued by the United States Preventive Services Task Force (USPSTF), previously fully screened women aged over 65 years old and presenting low-risk for cervical cancer are exempted from screening [6]. However, decreased immunity and the change of vaginal Microecology in postmenopausal women increases the risk of HPV infection, and negatively impacts its prognosis and clearance from the body, which makes it easier to develop into a persistent infection then causing cervical cancer. At the same time, with the aging of the population and the increasing number of menopausal population, the number of elderly patients with cervical cancer also increases correspondingly. Among them, women over 65 years old can account for 20% of the total number of new cases of cervical cancer [7]. However, women aged 65 to 69 who had cervical cancer screening in the first three years had a lower risk of dying from cervical cancer [8]. Thus it can be seen, cervical cancer screening for women over 69 years old can indeed reduce the mortality of cervical cancer. Therefore, the proposal to terminate the screening age has been controversial in recent years.

Due to has no consensus on when to terminate cervical cancer screening in postmenopausal women, and also there are few studies on screening for women over 65 years old. Hence, the age and conditions for withdrawal from screening are debatable. HPV is the main pathogenic factor of cervical cancer. Therefore, this study investigated the HPV distribution characteristics, infection rate, and risk of age in pre- and postmenopausal women who underwent cervical cytology, and HPV examination from January 2018 to October 2019 at Wuhan Union Hospital, so as to provide theoretical basis for the screening strategy of cervical cancer in postmenopausal women.

## Methods

### Target population

**Fig. 1:**
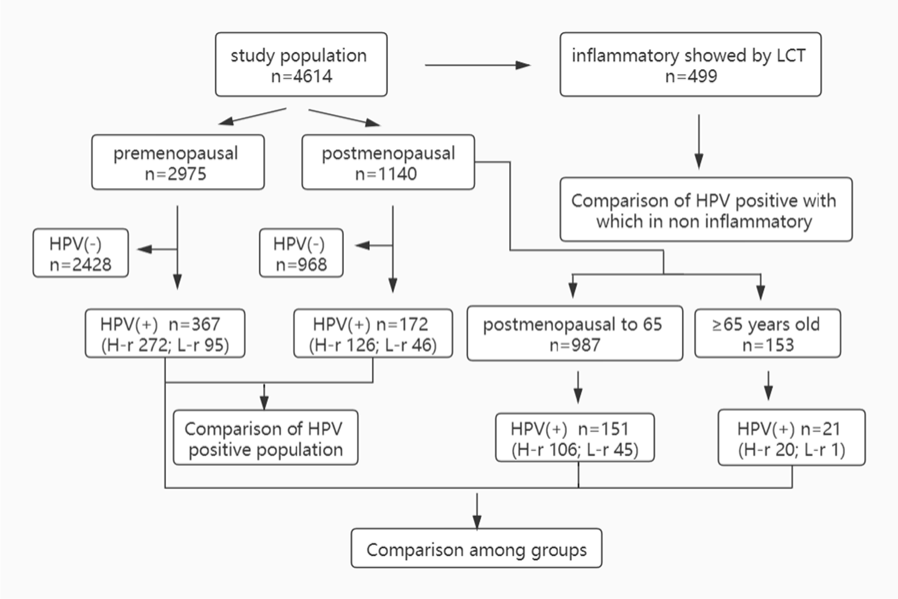
Flowchart of the study

In this study, 4614 women, aged 21–81 years (average 42.8 years old), who underwent cervical cytology and HPV examination at the healthcare department of Wuhan Union Hospital from January 2018 to October 2019 were selected. All the women had no conscious discomfort. Inclusion criteria: non menstrual women who have sex; asexual behavior within 24 h; no vaginal medication/irrigation recently. Exclusion criteria: cervical neoformation, hypertrophy, and glandular cyst; history of cervical laceration and operation; unsatisfactory quality of LCT smear, inflammatory results, visible endometrial cells; genital tract malformation. The studied population of women was categorized as premenopausal and postmenopausal based on their age. Furthermore, the characteristics of HPV infection in women aged over 65 years were examined. The flowchart is shown in Fig. 1.

### Experimental methodology

Cervical cytology using ultra cypress Liquid-based Cytologic Test (LCT) was employed to screen for cervical cancer and combined with HPV in the studied group of patients. Following LCT examination, 499 women were excluded due to bacterial, candida or trichomonas vaginalis infections; hence, 4115 women (2975 premenopausal and 1140 postmenopausal) women were eligible for HPV analysis. Classifing in the light of the diagnostic system of Bethesda (TBS) in 2014 [9], 17 high-risk HPV types (16/18/26/31/33/35/39/51/52/53/56/58/59/66/68/82), and 11 low-risk HPV types (06/11/40/42/43/44/55/61/81/83/73) were tested based on Luminex 200. Those with abnormal cervical cytology and positive for high-risk HPV were referred to gynecological clinic.

### Statistical analysis

The SPSS statistical software version 19.0 was used for analysis. In this study, the distribution of HPV types was count data, and the count was expressed by frequency and percentage (%). The chi-square test was used for comparison between groups. The risk of HPV infection was analyzed by single factor Logistic regression analysis. *P* < 0.05 was deemed statistically significant.

### Trial registration

Unregistered.

## Results

### HPV infection profiles and distribution

**Fig. 2:**
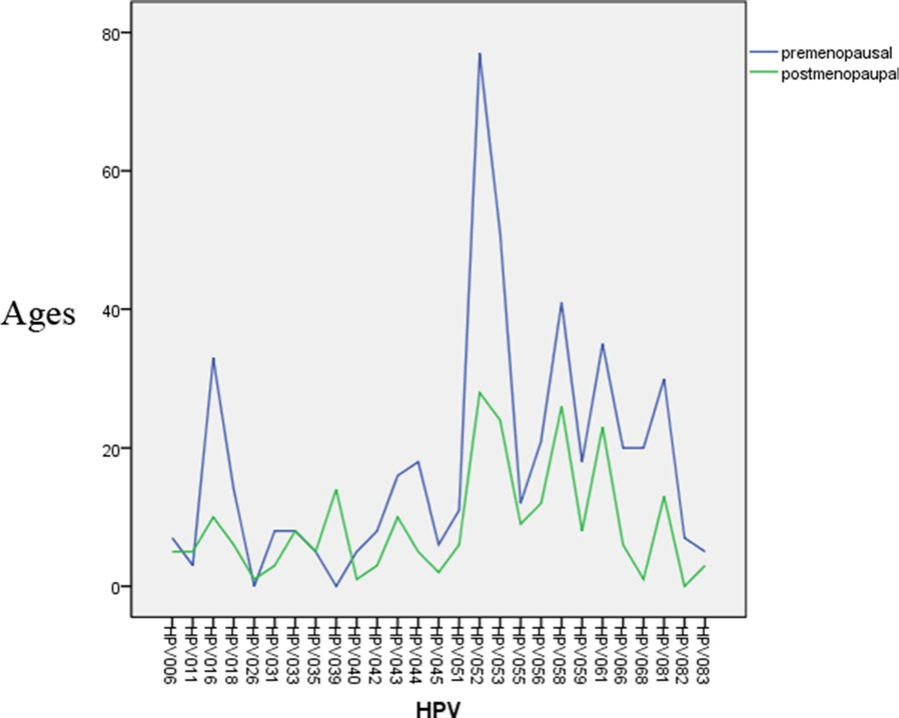
The frequency distribution of HPV infection

The frequency distribution of HPV infection before and after menopause is shown in Fig. 2. It can be seen that except HPV39 was more common in postmenopausal women, the spread of the other types were basically the same. Generally, 539 women test positive for HPV, representing 13.10% positive rate, mainly high risk types, which was 73.84% (398/539). The four leading HPV types in the studied population were HPV52, HPV53, HPV58, and HPV16, respectively. The frequency proportion of each high risk type is shown in Table 1.

**Table 1:** The frequency proportion of high risk HPV

	Premenopause		Postmenopause		HPV(+) total	Percentage (%)*
	−	+	−	+		
HPV16	2942	33	1130	10	43	1.04
HPV18	2961	14	1134	6	20	0.49
HPV26	2975	0	1139	1	1	0.02
HPV31	2967	8	1137	3	11	0.27
HPV33	2967	8	1132	8	16	0.39
HPV35	2970	5	1135	5	10	0.24
HPV39	2975	0	1126	14	14	0.34
HPV45	2969	6	1138	2	8	0.19
HPV51	2964	11	1134	6	17	0.41
HPV52	2898	77	1112	28	105	2.55
HPV53	2924	51	1116	24	75	1.82
HPV56	2954	21	1128	12	33	0.80
HPV58	2934	41	1114	26	67	1.63
HPV59	2957	18	1132	8	26	0.63
HPV66	2955	20	1134	6	26	0.63
HPV68	2955	20	1139	1	21	0.51
HPV82	2968	7	1140	0	7	0.17
HPV(+) Total	340		160		500	12.15

*Percentage of HPV positive frequency in total population

### Infection rate on HPV in women with inflammation

499 of 4614 physical examinees were found to be infected with bacteria, trichomonad or other inflammation. Through analysis, the results further revealed that the rate of HPV infection was significantly higher in women with inflammation than in those without inflammation (*P* = 0.000, 95% CI 1.911 (1.416, 2.580); Table 2).

**Table 2:** Comparison of HPV infection rate between women with and without inflammation

Inflammatory state	Total		HPV(+)		Percentage (%)		*P*	95% CI
	Pre-postmenopause		Pre-postmenopause		Pre-postmenopause			
No inflammation	2003	739	211	83	10.53	11.23	0.000	1.911 (1.416, 2.580)
Inflammation	367	132	63	21	17.17	15.91		

### The HPV infection in pre- and postmenopausal women

Besides, there was no significant difference between the infection rates in pre- and postmenopausal women (*P* = 0.056; Table 3), which were 34% (367/2975) and 15.09% (172/1140) respectively. Moreover, the postmenopausal population was further divided into two groups: postmenopausal to 65 years old and over 65 years old, comparing the three groups and discovered a markable difference in the infection rate among them (*P* = 0.041), then compared them in pairs, the results revealed that women aged over 65 years were significantly more susceptible to h-rHPV infection (*P* = 0.024/0.012; Tabe 3).

**Table 3:** Comparison of HPV infection rate between pre- and postmenopausal women

Age groups	Total (N)	HPV(+) (n)	n/N (%)	*P*	H-rHPV(+)	L-rHPV(+)	*P*	
Premenopausal	2975	367	12.34	0.056	272*	95	0.024	0.041
Menopausal to 65	987	151	15.09		106*	45	0.012	
Above 65	153	21			20	1	-	

*Compared with those over 65 years old separately

### The influence of h-rHPV infection in different age groups

The h-rHPV infection rate in the age bracket from 40 years old to menopause was differs from that in postmenopause, with remarkable diversity (*P* = 0.023, 0.729 (0.555, 0.957)); Table 4), and the rest age groups had nothing to do with h-rHPV infection.

**Table 4:** The influence of h-rHPV infection in different age groups

Age	n/N	Percentage (+)	*P*	95% CI
≤ 30	50/401	12.47	0.443	1.146 (0.808, 1.626)
30–40	117/1310	8.93	0.080	0.789 (0.605, 1.029)
40 to menopause	105/1264	8.31	0.023	0.729 (0.555, 0.957)
Postmenopause	126/1140	11.05	–	1
Menopause to 65	106/987	10.74	–	1
≥ 65*	20/153	13.07	0.393	0.800 (0.480, 1.334)

*Compared to menopausal to 65 years old

## Discussion

In healthy women, the normal microflora and local immune function of vagina along with host and environment constitute a micro ecosystem of mutual restriction, coordination and dynamic balance. When the balance is disturbed, women become more vulnerable to pathogenic infections, which cause various reproductive and urinary tract diseases. Under these conditions, the HPV invades and colonizes the genital tract, and its persistence causes cervical cancer [10, 11].

A meta-analysis involving more than 1 million women in 194 articles [12] showed that the total infection rate of HPV in women in different regions was 11.7% and the second peak of HPV infection occurred when they over 40, 45 or 55 years of age respectively. While, in the year 2005, a research on more than 8000 Costa Rican women reported that the infection rate of high-risk HPV beyond 65 years old was higher than that of 35–64 years old [3]. Our findings were basically consistent with above that. In this study, the total infection rate of HPV was 13.10%, mainly high-risk positive. There was no significant difference in the infection rate of HPV before and after menopause, but the high-risk subtype of HPV was more likely to be infected after the age of 65.

Furthermore, a previous study on HPV infection of 20,000 women in the nine provinces of China showed that the h-rHPV infection rate of more than 2000 postmenopausal women (17.2%) is not significantly different from that of non- menopausal women (16.4%) [13], which are consistent with those of Zhao et al. [14]. A study by Yang et al. [15] analyzed the data of cervical cancer screening in Wuxi and found a significantly higher HPV positive rate in aged 55–65 years. In a word, it did not make a distinction before and after menopause in the total HPV infection rate, but in a certain age group after menopause the infection rate is significantly higher than that in other age groups. The high HPV prevalence in postmenopausal women could be due to advancing age, gradual loss of ovarian function, wear thinning of vaginal mucosa, and lactobacilli reduction or even disappearance by degrees. However, the composition of vaginal microecology is significantly associated with HPV infection status [16]. The clearance rate of HPV and the risk of HPV related tumor are also closely correlation with the composition of vaginal flora [17]. Meanwhile, the gradual upward movement of the junction area between vagina and cervical squamous column, and the slow metaplasia of squamous epithelium. As a result, the reproductive system of postmenopausal women becomes more susceptible to carcinogenic factors and cancer-associated infections.

Elsewhere, various studies have indicated that the most common types of HPV are HPV16, HPV18, HPV52, and HPV58 [12, 18]; however, in our study, the most prevalent HPV types were HPV52, HPV53, HPV58, and HPV16. This inconsistency could be attributable to regional differences, and the varied pathogenicity among different HPV types. Practice guidelines recommend vaginal biopsy examination in women who test positive for HPV16 and HPV18 (important carcinogenic drivers) regardless of cervical cytology results [19].

This study also found that the infection rate of HPV in patients with genital tract inflammation was significantly higher than that in patients without inflammation. These results corroborate those of Meng and Yang [20, 21] who reported similar findings. It may be due to the changes of inflammatory factors in local microenvironment impair proper immune functioning, plus the vaginal defense mechanism is destroyed by pathogenic microorganisms, which also creates favorable conditions for the invasion and proliferation of pathogenic microorganisms, these all increasing the risk of HPV infection [11].

Current cervical cancer screening guidelines by the USPSTF (2018) suggest that women over 65 years old who have no previous high-risk factors and fully screened can be excluded for further examinations. However, a study researched by Castañón fond that the protective strength of previous negative screening results on women from cervical cancer weared off as time goes by; the number of patients who stopped screening at 65 years old was twice as much as that at 75 years old [22]. This study also showed that the HPV infection rate was significantly higher in women aged over 65 years.

However, China's National Cancer Center data demonstrate that new cases of cervical cancer in women aged over 60 years account for 23.76% of the total new cases, out of which 19.21% are 60–74-year-olds, and 4.55% are over 75 years old [23]. Therefore, the practice guidelines can only be used as guidance, not as a substitute for clinical decision-making. In clinical work, the advantages and disadvantages should be weighed, and personalized screening should be carried out.

There are still some deficiencies in this study. The sample size of women over 65 years old, especially HPV positive women over 65, is small, which may cause some deviation in the research results. Concurrently, the outcome of HPV infection in this kind of population can not be tracked, which reduces its guiding significance to a certain extent. The follow-up study is expected to make up for these defects and conduct in-depth discussion.

## Conclusions

This study shows that there is still a certain infection rate of h-r HPV after the age of 65, so we should reconsider the termination age of cervical cancer screening. On the other hand, there is no specific study on cervical cancer screening for women over 65 years old nowadays, so there is no direct evidence to determine the optimal screening age. With the increase of life expectancy in China, we should consider extending the age of termination of screening, and think over HPV typing as the primary screening method, which is of great significance for the prevention of cervical cancer in this group of people.

## Data Availability

The datasets generated and/or analysed during the current study are not publicly available due [The original data were presented in Chinese. Due to the large amount of data and the complexity of the content, it could not be translated into English in a short time, and a few patients were unwilling to disclose their personal data, so the original data were not published] but are available from the corresponding author on reasonable request.

## Abbreviations

HPV: Human papillomavirus
USPSTF: United States Preventive Services Task Force
TBS: The Bethesda System
LCT: Liquid-based Cytologic Test
H-rHPV: High-risk HPV
